# The Use of Portal Vein Pulsatility to Differentiate Hypervolemic and Hypovolemic Hyponatremia

**DOI:** 10.1155/2019/9591823

**Published:** 2019-07-15

**Authors:** Gurkeerat Singh, Jean-Sebastien Rachoin, Christina Chien, Sharad Patel

**Affiliations:** Critical Care, Cooper University Hospital, USA

## Abstract

Hypotonic hyponatremia is a common electrolyte disorder defined by a blood serum sodium value of less than 136 meq/L. A challenge in managing hyponatremia is accurately determining the etiology for the free water excess as management can markedly differ. Accurate diagnosis of the etiology of hypotonic hyponatremia requires precision in the determination of extracellular volume status. Determination of volume status has traditionally relied on physical examination, imaging modalities, and clinical gestalt, all of which are inaccurate. Portal vein pulsatility is an easy to perform bedside ultrasound imaging study which can be used as a marker for hypervolemia and venous congestion. We present 2 cases of hypervolemic hyponatremia in which portal vein pulsatility was used in the diagnosis and management and as a marker for efficacy of treatment.

## 1. Introduction

Hypotonic hyponatremia is a pervasive electrolyte disorder that represents an excess of free water as compared to sodium [1]. Conditions that lead to reduced free water clearance predispose to hyponatremia. Reductions in free water clearance can occur in situations with either inappropriate Antidiuretic Hormone (ADH) excess and or neurohormonal activation in settings of a low effective circulating volume state. Hypotonic hyponatremia can be further broken down by extracellular volume such as hypovolemic, euvolemic, and hypervolemic states. Precision in the diagnosis of volume status is notoriously poor when dependent on the traditional examination findings of pitting edema and diagnostic studies such as chest X-ray, which can lead to inappropriate interventions. Urine electrolytes can be helpful but due to analogous neurohormonal activation related proximal tubular sodium retention hypovolemic and hypervolemic hyponatremia will exhibit similar urine sodium values. We introduce the use of portal vein pulsatility to delineate the difference between hypovolemic and hypervolemic hypotonic hyponatremia, specifically in conditions of low effective circulating volume (ECV) due to congestive heart failure. Denault et al. introduced the use of portal vein pulsatility as a marker for renal vein congestion which correlated with acute kidney injury (AKI) in postcardiac patients [2]. Renal venous congestion leads to increased neurohormonal activation with a concurrent increase in ADH both of which can contribute to hypervolemic hyponatremia (for the purposes of this paper we will use the term venous congestion to indicate hypervolemia and vice versa). Persistent neurohormonal activation from venous congestion will eventually lead to AKI [3]. Venous congestion related hyponatremia and AKI can be considered a spectrum of renal insults; therefore it is logical to conclude that portal vein pulsatility can provide guidance for hypervolemic hyponatremia. We present two cases where portal vein pulsatility was used to diagnose and manage congestive heart failure related hyponatremia.

## 2. Case 1

**Figure 1:**
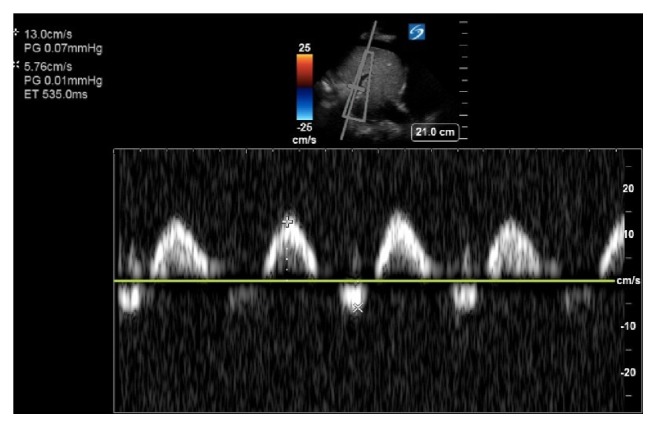
Portal vein Doppler exam performed 4hrs after ROSC on day 1. There is a biphasic portal vein profile with flow reversal. Maximum and minimum velocities are 13 and 5.76 cm/s, respectively. Pulse is 85 beats per minute (bpm). Portal vein pulsatility fraction is greater than 100 %. Ascites is present.

**Figure 2:**
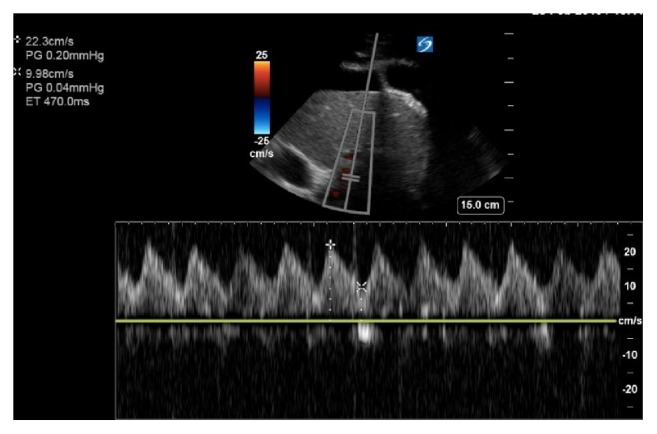
Portal vein Doppler exam on day 3. Maximum and minimum velocities are 22.3 and 9.98 cm/s, respectively. Pulse is 102 bpm. Portal vein pulsatility fraction is 59 %. Ascites is once again noted.

A 54-year-old male presented to the emergency department after a pulseless electrical activity (PEA) cardiac arrest at home with an estimated downtime of 20 minutes. He received a total of 5 mg of IV epinephrine, 2 mg of IV magnesium, and 200 mEq of sodium bicarbonate. After achieving a return of spontaneous circulation (ROSC), the hypothermia protocol was initiated, and the patient was transferred to the intensive care unit. He was on mechanical ventilation with an initial fraction of inspired oxygen requirement of 100% on a positive end-expiratory pressure of 12 cm H20. He was on norepinephrine of 20 mcg/min to maintain a mean arterial pressure of greater than 65 mmHg. Initial labs were notable for a white blood count of 19000 cells per cubic millimeter (4.5-11000), sodium 115 meq/L (135-145 meq/L), and a N-terminal pro b-type natriuretic peptide of 12100 pg/ml (1-850 pg/ml Table 1). Urine sodium was less than five meq/L and calculated fractional excretion of sodium was less than 1 %. Abdominal CT scan did not show any features suggestive of cirrhosis. Bedside point of care ultrasound was remarkable for a moderately reduced biventricular function; a significantly dilated inferior vena cava and a high portal vein pulsatility fraction (Figure 1). Diuresis was initiated by starting IV furosemide 60 mg every 8 hours with concurrent metolazone 5 mg every 12 hours. A negative 3.5 L volume state was achieved over the next 48 hours. The patient's sodium corrected at an appropriate rate, with a total increase in 16 meq (115 to 131 meq/L) in 48 hours. Our team was careful in abiding by the recommendations to avoid overcorrection with goal sodium improvements of less than ten meq in 24 hours and less than 18 meq over 48 hours. Repeat bedside portal vein assessment (Figure 2) showed a substantial decrease in portal vein pulsatility fraction. With the negative fluid balance, the patient's hemodynamics and ventilator requirements improved as well. Despite cardiopulmonary improvements, the patient's neurologic exam remained poor 72 hours after targeted temperature management, and the family elected to make the patient comfort care.

## 3. Case 2

**Figure 3:**
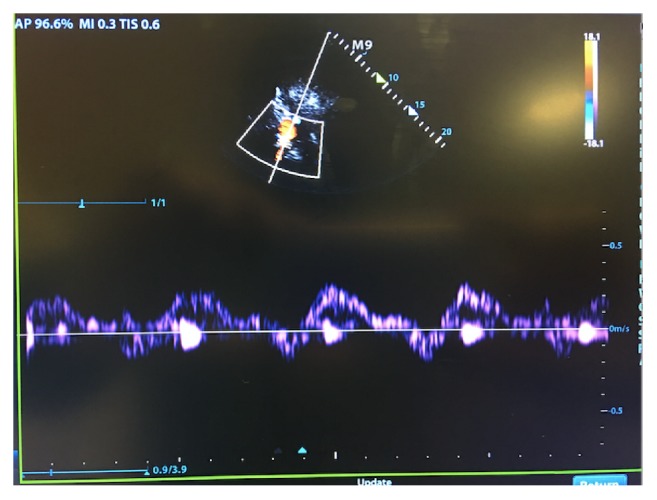
Biphasic portal vein Doppler profile with flow reversal performed on day 1. Pulsatility fraction more than 100 % with heart rate at 95 bpm.

**Figure 4:**
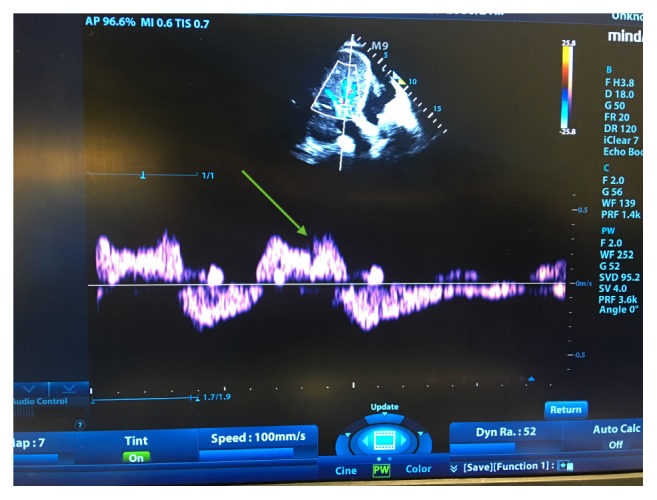
Hepatic vein Doppler exam on day 1. Evidence of systolic flow reversal (green arrow).

**Figure 5:**
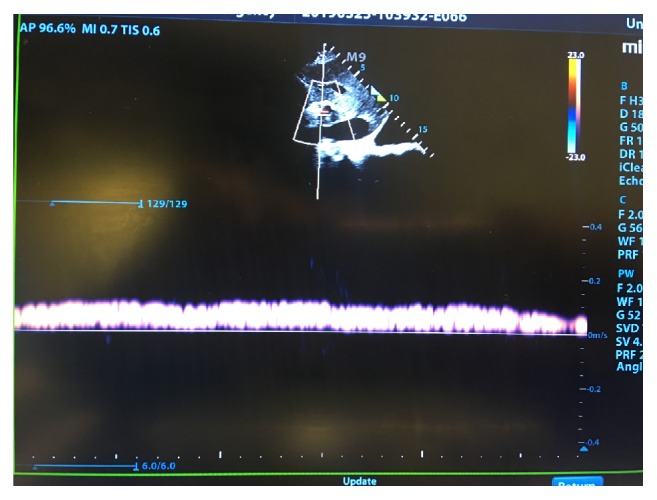
Portal vein Doppler exam on day 3. The pulse was 90 bpm. The pulsatility fraction was less than 20%.

A 95-year-old male with a past medical history of heart failure with reduced ejection fraction, atrial fibrillation, and hypothyroidism presented to the hospital with shortness of breath and increased work of breathing requiring noninvasive positive pressure ventilation. Laboratory values were remarkable for a serum sodium of 120 meq/L, serum creatinine of 1.91 mg/dl, and an NT ProBNP above 15000 pg/ml (Table 2). Bedside portal vein assessment showed a biphasic portal vein pattern consistent with venous congestion (Figure 3). Hepatic vein Doppler was also suggestive of volume congestion (Figure 4). The clinical picture was likely consistent with venous congestion related AKI and hyponatremia. Diuresis with IV furosemide and PO metolazone was initiated, leading to a net negative fluid balance of 3.5 L in the next 48 hours. Significant improvement in symptoms, serum sodium levels (120 to 136 meq/L), and a decrease in the portal vein pulsatility fraction (Figures 3 and 5) was also seen. The patient was discharged home on day 5.

## 4. Discussion

Hyponatremia secondary to congestive heart failure is a prognostic marker for mortality [4]. Congestive heart failure can lead to significant neurohormonal activation leading to an increase in both free water and sodium retention predisposing to hyponatremia. As ECF is reduced, there will be baroreceptor activation with a subsequent increase in renin-angiotensin (RAAS), sympathetic nerve innervation, and ADH, which leads to hypervolemia, hyponatremia, and eventual AKI. This neurohormonal activation is an adaptive evolutionary mechanism that was protective in states of hypovolemia but can be maladaptive in congestive heart failure. Despite a condition of increased total body sodium, the response to the low ECF is avid proximal tubule sodium retention, which further exacerbates overall organ congestion.

Urine electrolytes in case 1 indicates a sodium avid state, as the urine sodium value was < 5, which indicates a low ECF, and does not differentiate hypervolemia or hypovolemia. The initial portal vein study was biphasic in both cases, which denotes severe venous congestion and more consistent with hypervolemia. Once a diagnosis of hypervolemic hyponatremia was made, we felt that achieving a negative fluid balance would lead to an improvement in renal venous congestion with possible concurrent renal interstitial edema and intra-abdominal hypertension. We were able to demonstrate significant improvements in pulsatility that coincided with the improvements in sodium as we diuresed the patients. Both patients either maintained or improved their creatinine levels with diuresis that was guided by improvements in pulsatility. We believe that following portal vein pulsatility improvements could potentially avoid acute kidney injury from overdiuresis.

**Figure 6:**
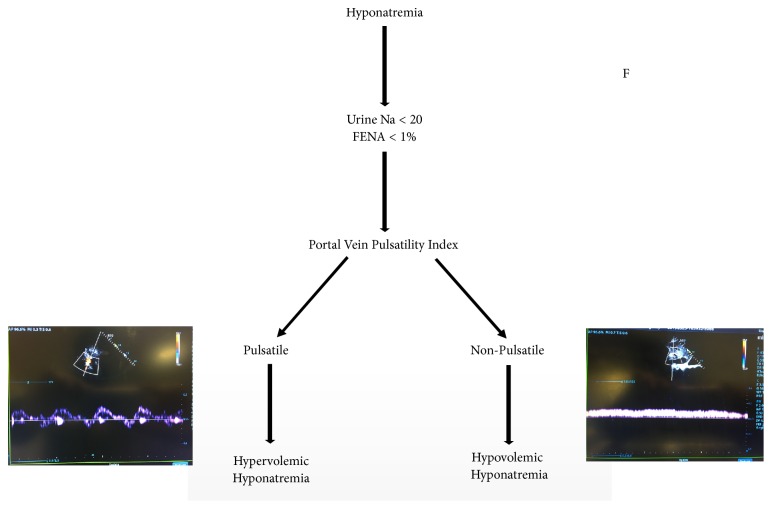
Proposed algorithm.

The use of portal vein pulsatility to identify patients with hypervolemic hypotonic hyponatremia would portend an essential advance in the diagnosis of the condition, which has mostly remained unchanged for decades. Differentiation between hypervolemia and hypovolemia, particularly when it comes to venous congestion, remains challenging with the traditional methods of physical exam and chest X-ray. Furthermore, repeat measurements of pulsatility allow for the clinician to assess for improvements in organ congestion, potentially avoiding excessive diuresis. Prior studies have demonstrated a high success rate and good interobserver variability which bolster the case for the more pervasive use of portal vein pulsatility in the management of hyponatremia [5]. A proposed algorithm is presented in Figure 6.

There remain significant limitations to our proposal for the use of portal vein pulsatility to diagnose and follow therapeutic efficacy for hypervolemic hyponatremia. First, though the physiology is viable, there needs to be a more extensive proof of concept study performed, as we are still in the hypothesis phase. Secondly, though prognosis appears to be worse in heart failure patients with hyponatremia, there is little evidence that modification of the hyponatremia would improve mortality outcomes. Instead, the goal is to improve symptoms and morbidity; for example, sodium levels below 120 meq/L can alter mentation and predispose to seizures, and correction can improve these outcomes.

## 5. Conclusions

We present two cases of hypervolemic hyponatremia. Hypervolemic hypotonic hyponatremia is both challenging to diagnose and manage. We propose portal vein pulsatility as a means to diagnosis and as an indicator for efficacy of therapeutics. The study is relatively simple to perform with excellent interobserver variability. Our case series is hypothesis generating, and a more extensive study will need to be performed to further corroborate the use of portal vein pulsatility index in the management of hypervolemic hyponatremia.

## Figures and Tables

**Table 1:** 

	Na	K	Cl	BUN	Cr	CO2	NT-ProBNP
Day 1	115 meq/L	5.3 meq/L	88 meq/L	19 mg/dl	0.8 mg/dl	12 meq/L	12100 pg/ml
Day 3	131 meq/L	3.0 meq/L	100meq/L	11 mg/dl	0.5 mg/dl	31 meq/L	

Na: sodium, K: potassium, Cl: chloride, BUN: blood urea nitrogen, Cr: creatinine, CO2: blood carbon dioxide, and NT-ProBNP: N-terminal pro b-type natriuretic peptide.

**Table 2:** 

	Na	K	Cl	BUN	Cr	Co2	NT-pro-BNP
Day 1	120 meq/L	6.1 meq/L	88 meq/L	55 mg/dl	1.91mg/dl	15 meq/L	15061 pg/ml
Day 3	136 meq/L	4.6 meq/L	86 meq/L	62 mg/dl	1.53 mg/dl	32 meq/L	

Na: sodium, K: potassium, Cl: chloride, BUN: blood urea nitrogen, Cr: creatinine, CO2: blood carbon dioxide, and NT-ProBNP: N-terminal pro b-type natriuretic peptide.
